# Associations Between Using Chinese Herbal Medicine and Long-Term Outcome Among Pre-dialysis Diabetic Nephropathy Patients: A Retrospective Population-Based Cohort Study

**DOI:** 10.3389/fphar.2021.616522

**Published:** 2021-02-18

**Authors:** Jenny Chun-Ling Guo, Heng-Chih Pan, Bo-Yan Yeh, Yen Chu Lu, Jiun-Liang Chen, Ching-Wei Yang, Yu-Chun Chen, Yi-Hsuan Lin, Hsing-Yu Chen

**Affiliations:** ^1^ Division of Chinese Internal Medicine, Center for Traditional Chinese Medicine, Chang Gung Memorial Hospital, Taoyuan, Taiwan; ^2^ College of Medicine, Chang Gung University, Taoyuan, Taiwan; ^3^ Division of Nephrology, Department of Internal Medicine, Keelung Chang Gung Memorial Hospital, Keelung, Taiwan; ^4^ Division of Acupuncture and Moxibustion, Department of Traditional Chinese Medicine, Chang Gung Memorial Hospital, Taoyuan, Taiwan; ^5^ School of Traditional Chinese Medicine, College of Medicine, Chang Gung University, Taoyuan, Taiwan; ^6^ School of Medicine, Faculty of Medicine, National Yang Ming Chiao Tung University, Taipei, Taiwan; ^7^ Graduate Institute of Clinical Medical Sciences, College of Medicine, Chang Gung University, Taoyuan, Taiwan

**Keywords:** Chinese herbal medicine, diabetic nephropathy, end-stage renal disease, mortality, network analysis, pre-dialysis

## Abstract

**Background:** Chronic kidney disease (CKD) has become a worldwide burden due to the high co-morbidity and mortality. Diabetic nephropathy (DN) is one of the leading causes of CKD, and pre-dialysis is one of the most critical stages before the end-stage renal disease (ESRD). Although Chinese herbal medicine (CHM) use is not uncommon, the feasibility of using CHM among pre-dialysis DN patients remains unclear.

**Materials and methods:** We analyzed a population-based cohort, retrieved from Taiwan’s National Health Insurance Research Database, to study the long-term outcome of using CHM among incident pre-dialysis DN patients from January 1, 2004, to December 31, 2007. All patients were followed up to 5 years or the occurrence of mortality. The risks of all-cause mortality and ESRD were carried out using Kaplan-Meier and competing risk estimation, respectively. Further, we demonstrated the CHM prescriptions and core CHMs using the Chinese herbal medicine network (CMN) analysis.

**Results:** A total of 6,648 incident pre-dialysis DN patients were analyzed, including 877 CHM users and 5,771 CHM nonusers. With overlap weighing for balancing all accessible covariates between CHM users and nonusers, we found the use of CHM was associated with lower all-cause mortality (0.22 versus 0.56; log-rank test: *p*-value <0.001), and the risk of mortality was 0.42 (95% CI: 0.36–0.49; *p*-value <0.001) by adjusting all accessible covariates. Further, the use of CHM was associated with a lower risk of ESRD (cause-specific hazard ratio: 0.59, 95%CI: 0.55–0.63; *p*-value <0.001). Also, from the 5,901 CHM prescriptions, we found Ji-Sheng-Shen-Qi-Wan, *Astragalus mongholicus* Bunge or (*Astragalus membranaceus* (Fisch.) Bge.)*, Plantago asiatica* L. (*or Plantago depressa* Willd.), *Salvia miltiorrhiza* Bunge, and *Rheum palmatum L.* (*or Rheum tanguticum* (Maxim. ex Regel) Balf.*, Rheum officinale* Baill.) were used as core CHMs for different CHM indications. Use of core CHMs was associated with a lower risk of mortality than CHM users without using core CHMs.

**Conclusions:** The use of CHM seemed feasible among pre-dialysis DN patients; however, the beneficial effects still need to be validated by well-designed clinical trials.

## Introduction

Chronic kidney disease (CKD) has become a worldwide burden due to the high co-morbidity and mortality (Bikbov et al., 2020). CKD has been thought of as a syndrome and may originate from various etiologies (Vallon and Komers, 2011). Diabetic nephropathy (DN) is one of CKD's leading causes, accounting for 20–30% of CKD patients (Soldatos and Cooper, 2008). Twenty patients will be diagnosed with end-stage renal disease (ESRD) in every 10,000 diabetes mellitus (DM) patients each year, according to the U.S. Renal Data System report in 2010 (Gregg et al., 2014). On the other hand, pre-dialysis is one of the most critical stages before the occurrence of ESRD (Singhal et al., 2014); nonetheless, only some medications were studied about the safety and even benefits for CKD patients in the pre-dialysis status, such as renin-angiotensin-aldosterone system (RAAS) blockage agents and ketoanalogues supplements (Wu et al., 2013; Li et al., 2019a). The information is far less when speaking of pre-dialysis DN patients.

On the other hand, although traditional Chinese medicine (TCM) use, including Chinese herbal medicine (CHM), acupuncture, or tuina, is not uncommon among Asian populations, including Taiwan, the feasibility of using CHM among pre-dialysis CKD patients remains unclear. Most clinical and bench studies showed that the use of TCM might be beneficial for CKD patients but lacking in the information of specified stages or long-term outcomes (Chen et al., 2019). Previous studies proved that CHM might be useful in improving renal function and delaying the starting time of renal replacement therapy. For example, Liu-Wei-Di-Huang-Wan, a decoction containing six CHMs, delayed 1 year of kidney failure development in type 2 DM patients (Hsu et al., 2014a). Lin et al. suggested that CHM may have reno-protective effects in CKD patients before the occurrence of ESRD (Lin et al., 2015). Also, an analysis based on hospital data concluded that Jia-Wei-Xiao-Yao-San (JWXYS) and Bu-Yang-Huan- Wu-Tang (BYHWT) were the top two decoctions prescribed to CKD patient and could stabilize renal function decline (Yang et al., 2014; Chen et al., 2018). Furthermore, a systematic review and meta-analysis research that included 20 studies with 2,719 patients from nine databases shows that albuminuria improved under CHM use and estimated glomerular filtration rate (eGFR) was better using CHM along with RAAS blockers without analyzing the long-term outcomes (Zhang et al., 2019).

We previously reported that TCM treatments might be associated with better outcomes among all DN patients without knowing the feasibility of using CHM among pre-dialysis DN patients (Chen et al., 2019). This study aims to assess the associations of CHM use with the long-term outcome among incident pre-dialysis DN patients, such as all-cause mortality and the occurrence of ESRD. Besides, the prescriptions made for the pre-dialysis DN patients were analyzed to disclose the core CHMs used for these patients.

## Materials and Methods

### Data Source

All data were obtained from the National Health Insurance Research Database (NHIRD), a population-based clinical database in Taiwan famous for its high integrity and is commonly used for epidemiological studies (Chen et al., 2012; Wu et al., 2012; Chang et al., 2018; Hsieh et al., 2019). TCM is fully reimbursed in Taiwan, while two kinds of CHM were approved: single herb (SH) and herbal formula (HF). HFs are composed of SHs in a classic and theoretical manner corresponding with CHM prescription theory, working together as monarch, minister, assistant, and guide, for example, Jia-Wei-Xiao-Yao-San (JWXYS) and Bu-Yang-Huan- Wu-Tang (BYHWT) mentioned in this study. On the other hand, SHs, as the name implies, are prepared from single material recorded in the CHM classics, such as *Astragalus mongholicus* Bunge*, Plantago asiatica* L., *Salvia miltiorrhiza* Bunge, and *Rheum palmatum* L.. HFs are pre-mixed before marketing, and therefore TCM doctors can combine HFs or SHs freely based on each patient’s condition.

### Study Designs and Ethical Considerations

**FIGURE 1:**
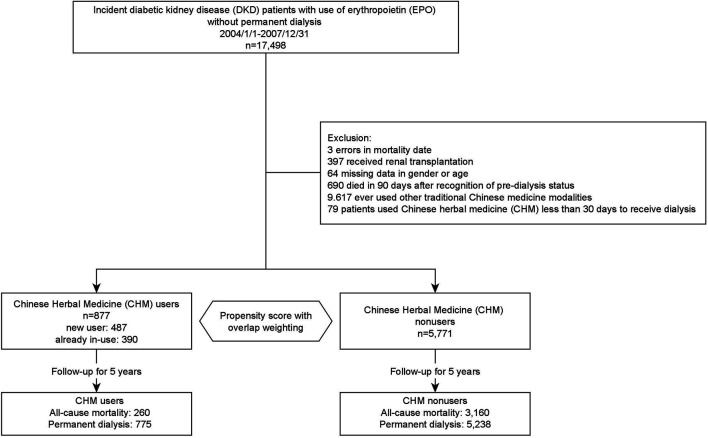
Flow diagram of this study.

Figure 1 illustrates the research protocol and data processing flow. From 2004/1/to 2007/12/31, type 2 DM patients with two consecutive diagnoses of CKD in out-patient services or one diagnosis in hospitalization after that were defined as having DN (Chen et al., 2019). The International Classification of Diseases, 9th Revision, Clinical Modification (ICD-9-CM) codes were used to recognize these patients (type 2 DM: 250.x, except 250.x1 and 250.x3; CKD: 580.x-588.x, 250.4x, 274.1x, 283.11, 403.x1, 404.x2, 404.x3, 440.1, 442.1, 447.3, 572.4, 642.1x, 646.2x and 791.0). Furthermore, we used the initiation of prescribing EPO as the recognition of pre-dialysis DN patients (Hsu et al., 2014b; Lin et al., 2017). The initiation of using erythropoietin (EPO) without receiving permanent dialysis was regarded as pre-dialysis status because EPO is only approved for stage 5 CKD pre-dialysis patients with a hematocrit level <28% and serum creatinine level >6 mg/dl or patients receiving permanent dialysis therapy in Taiwan (Hsu et al., 2014b). Patients were included if diagnosed with incident diabetic nephropathy (DN) with EPO but without permanent dialysis from 2004/1/1 to 2007/12/31. CHM users were recognized by whoever used CHM for DN during the pre-dialysis status. On the other hand, patients were excluded if data were missing/error in gender, age, or mortality date, he or she ever received renal transplantation, or ever used other TCM modalities, such as acupuncture or tuina. Subjects who used CHM less than 30 days before getting dialysis were excluded as well. No consent was needed because the patients' identification number was encrypted, and therefore it is impossible to trace patients from the database. The research proposal was reviewed and approved by the Institutional Review Board (IRB) of the Chang-Gung Memorial Foundation (IRB No. 202001791B1).

### Outcome Assessment

Our study's primary outcome was all-cause mortality, and the occurrence of permanent dialysis, presenting the occurrence of ESRD, was set as the secondary outcome. The date for applying catastrophic illness certificates of permanent dialysis was regarded as the start of ESRD. In Taiwan, the certificate has to be applied by the nephrologists for the patients when their renal function is not expected to recover for at least 3 months, and therefore it becomes a reliable proxy to recognize patients entering ESRD (Wen et al., 2008; Chen et al., 2019). Every subject was followed for 5 years or until the deceased. To assess the duration effect, the use of CHM was further divided into two groups upon CHM duration: using CHM for over or less than 60 days.

### Study Covariates

Gender, age, co-morbidities, insured level, place of residence, and concurrently used medications were obtained from the NHIRD as covariates in this study. The insured level was divided into three levels, reflecting the income since premium depends on earnings. Co-morbidities potentially related to outcomes were investigated, such as hypertension, hyperlipidemia, heart failure, coronary artery disease (CAD), cerebrovascular disease (CVD), hyperuricemia, or cancer. Following medication use was extracted as well: insulin analogs, oral hypoglycemia agents (OHA), statin/fibrate for hyperlipidemia, angiotensin-converting enzyme inhibitor (ACEi)/angiotensin II receptor blocker (ARB), other anti-hypertensive drugs, nonsteroidal anti-inflammatory drug (NSAID), cyclooxygenase-2 (COX-2) inhibitor, acetaminophen, and aspirin. As the representative of socio-economic status, the insured levels and residence places were acquired for every patient and divided into 3 and 2 separately as previous works (Lin et al., 2017; Chen et al., 2019).

### Bias Assessment

Data obtained from the nationwide clinical database (NHIRD) contains the whole population that prevented selection bias and referral bias. The possible recall bias noted from study data obtained through questionnaires retrospectively could be eliminated since every record of admission, out-patient visits, and management had been continuously and automatically updated in the NHIRD in the prospective manner (Sedgwick, 2012). Detection bias was minimized since EPO's precise regulation for these subjects to be covered by the national health insurance program. Furthermore, patients would only be included if they survived more than 90 days as landmark time during the follow-up period to avoid immortal bias (Suissa, 2007).

### Statistical Analysis

Two parts of the analysis were conducted in this study: outcomes assessment and the Chinese herbal medicine network (CMN) analysis. Baseline demographic features were presented either as mean with standard deviation for continuous variables and count with percentage for categorical variables; Student’s *t*-test and *X* ^2^ statistics were used to compare the differences between CHM users and nonusers. Propensity score with overlap weighting was used to balance CHM users' and nonusers' baseline status and obliterate the imbalanced case numbers (Li et al., 2017). All viable demographic features in the database, such as age, gender, co-morbidities, and medications, were used to generate propensity scores with overlap weights for using CHM. With overlap weighting, Kaplan-Meier estimation and Cox regression were used to calculate the accumulative probability and hazard ratio (HR) of mortality, in which all accessible covariates were used to adjust the HR in the Cox regression model. Multivariate cox regression stratified by covariates and sensitivity tests were done to confirm the associations between using CHM and the all-cause mortality. Models with different covariates balancing methods (1:1 propensity score matching and the inverse-probability-of-the-treatment-weighting [IPTW] method) and different populations (with 180-day landmark analysis, without excluding patients who died with 90 days after the recognition of pre-dialysis, and without excluding patients with other TCM modalities) were used as sensitivity tests in this study.

Additionally, analysis of the CMN could reveal the treatment principle and core CHM for pre-dialysis DN, and we only included the patients using CHM for pre-dialysis DN. By excluding patients who ever used other TCM modalities, we could avoid the influences on prescription analysis caused by CHM prescriptions not indicating for DN or DM, but for related symptoms. The build-up of CMN was described extensively in our previous reports (Chen et al., 2015). Association rule mining was used to find out the common CHM combinations, and social network analysis was used to graphically demonstrate and analyze the CMN (Chen et al., 2015). Commonly used CHM were clustered according to the relations between CHMs, and core CHM could be found as the CHM with high prevalence and high degree centrality, meaning other CHMs were used when core CHM was prescribed. The associations between core CHMs and all-cause mortality were also examined individually. Stata (StataCorp. 2019. Stata Statistical Software: release 16. College Station, TX: StataCorp LLC) and NodeXL were used to perform the analysis in this study, and the statistics with *p*-value < 0.05 was regarded as significant results.

## Results

### Baseline Demographic Features

From 2004/1/1 to 2007/12/31, a total of 6,648 incident pre-dialysis DN patients were analyzed in the final stage of our study. Table 1 summarizes all analyzed subjects' baseline demographic features, in which 877 CHM users and 5,771 nonusers were identified. There were more men (54.6%) and elderly patients age more than 70 years old (57.4%) among CHM nonusers compared to CHM users, with 45.2% of men and 29% of patients over 70 years old (*p*-value < 0.001). For underlying co-morbidities, CHM nonusers had about 8–13% higher rates of hypertension versus nonusers, heart failure, CAD, and CVD than CHM users (*p*-value all <0.001), while no significant differences were noted on hyperlipidemia, hyperuricemia, and malignancies between the two groups. The percentages of using insulin, OHAs, Statin, other anti-hypertensive agents, and aspirin of CHM nonusers were higher, while no significant difference in using NSAIDs, acetaminophen, and COX-2 inhibitors (Table 1).

**TABLE 1:** The baseline demographic features of Chinese herbal medicine (CHM) users and nonusers.

	All patients (*n* = 6,648)		CHM nonusers (*n* = 5,771)		CHM users (*n* = 877)		*p*-value
Gender							
Female	3,099	(46.6%)	2,618	(45.4%)	481	(54.8%)	<0.001
Male	3,549	(53.4%)	3,153	(54.6%)	396	(45.2%)	
Age							
<70	3,937	(59.2%)	3,314	(57.4%)	623	(71.0%)	<0.001
≥70	2,711	(40.8%)	2,457	(42.6%)	254	(29.0%)	
Co-morbidities							
Hypertension	5,667	(85.2%)	4,994	(86.5%)	673	(76.7%)	<0.001
Hyperlipidemia	2,041	(30.7%)	1,787	(31.0%)	254	(29.0%)	0.23
Heart failure	1,657	(24.9%)	1,543	(26.7%)	114	(13.0%)	<0.001
CAD	1,844	(27.7%)	1,663	(28.8%)	181	(20.6%)	<0.001
CVD	954	(14.4%)	886	(15.4%)	68	(7.8%)	<0.001
Hyperuricemia	1,009	(15.2%)	892	(15.5%)	117	(13.3%)	0.10
* Cancer*	823	(12.4%)	719	(12.5%)	104	(11.9%)	0.62
Medications							
Insulin analogs	1,799	(27.1%)	1,663	(28.8%)	136	(15.5%)	<0.001
OHAs	3,796	(57.1%)	3,416	(59.2%)	380	(43.3%)	<0.001
Statin/Fibrate	2,614	(39.3%)	2,320	(40.2%)	294	(33.5%)	<0.001
ACEi/ARB	4,183	(62.9%)	3,689	(63.9%)	494	(56.3%)	<0.001
Other anti-hypertensive agents	6,172	(92.8%)	5,430	(94.1%)	742	(84.6%)	<0.001
NSAIDs	1,213	(18.2%)	1,047	(18.1%)	166	(18.9%)	0.57
COX-2 inhibitors	177	(2.7%)	153	(2.7%)	24	(2.7%)	0.88
Acetaminophen	1,400	(21.1%)	1,198	(20.8%)	202	(23.0%)	0.12
Aspirin	2,267	(34.1%)	2,045	(35.4%)	222	(25.3%)	<0.001
Insured level (NTD/month)							
0–20,000	5,850	(88.0%)	5,138	(89.0%)	712	(81.2%)	<0.001
20,001–40,000	529	(8.0%)	412	(7.1%)	117	(13.3%)	
40,001-	269	(4.0%)	221	(3.8%)	48	(5.5%)	
Place of residence							
More rural	2,830	(42.6%)	2,469	(42.8%)	361	(41.2%)	0.37
More urban	3,818	(57.4%)	3,302	(57.2%)	516	(58.8%)	

*Abbreviations: ACEi, angiotensin-converting enzyme inhibitor; ARB, angiotensin II receptor blocker; COX-2, cyclooxygenase-2; CAD, coronary artery disease; CVD, Cerebrovascular disease; OHAs, oral hypoglycemic agents; NSAID, nonsteroidal anti-inflammatory drug; NTD, new Taiwan dollar.*

### Use of CHM Associated with Lower 5-Year Mortality and the Occurrence of ESRD

**FIGURE 2:**
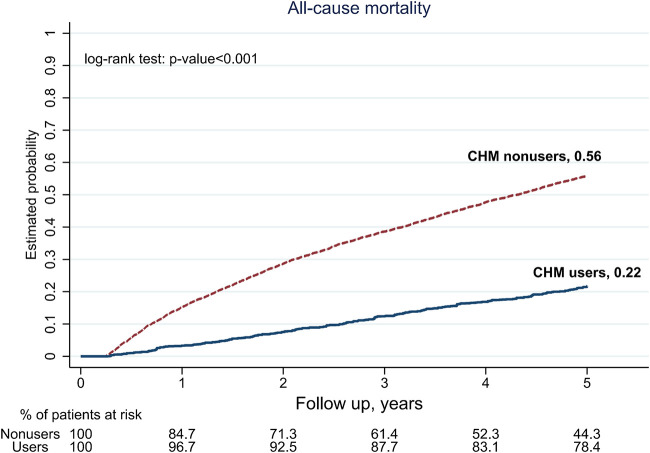
5-year follow-up for all-cause mortality among pre-dialysis diabetic nephropathy (DN) patients, by Chinese herbal medicine (CHM) users and nonusers.

At the end of the 5-year follow-up, a total of 3,420 subjects were deceased among 6,648 subjects, finally analyzed, in which 260 subjects were CHM users, and 3,160 subjects were CHM nonusers (Figure 1). With overlap weighting to balance the differences of all covariates, the use of CHM was associated with a lower probability of all-cause mortality than CHM nonusers (0.22 versus 0.56, by Kaplan-Meier estimation; log-rank test: *p*-value < 0.001) (Figure 2). While considering all accessible covariates, the risk of the all-cause mortality was still lower among CHM users (adjust hazard ratio [aHR]: 0.42, 95% CI: 0.36–0.49, *p*-value < 0.001, Table 2). Furthermore, this result had a duration-dependent effect. The risks of all-cause mortality reduced while increasing the duration of use of CHM, and the tendency remained consistent in the models either with or without overlap weighting and adjusting the demographic covariates (Table 2).

**TABLE 2:** Risks of all-cause mortality among Chinese herbal medicine (CHM) users, concerning the accumulative duration of CHM.

	Without overlap weighting				With overlap weighting			
	HR (95%CI)	*p*-value	aHR (95%CI)	*p*-value	HR (95%CI)	*p*-value	aHR (95%CI)	*p*-value
All CHM users	0.43 (0.38–0.49)	<0.001	0.53 (0.47–0.60)	<0.001	0.30 (0.25–0.34)	<0.001	0.42 (0.36–0.49)	<0.001
Use of CHM by accumulated duration (days)								
0 (*n* = 5,771)	1 (References)		1 (References)		1 (References)		1 (References)	
1–60 (*n* = 651)	0.47 (0.41–0.54)	<0.001	0.58 (0.51–0.67)	<0.001	0.33 (0.28–0.39)	<0.001	0.47 (0.40–0.56)	<0.001
61- (*n* = 226)	0.33 (0.25–0.43)	<0.001	0.39 (0.30–0.50)	<0.001	0.17 (0.12–0.25)	<0.001	0.25 (0.18–0.36)	<0.001

*Abbreviations: aHR, adjusted hazard ratio; CHM, Chinese herbal medicine; CI, confidence interval; HR, hazard ratio.*

**FIGURE 3:**
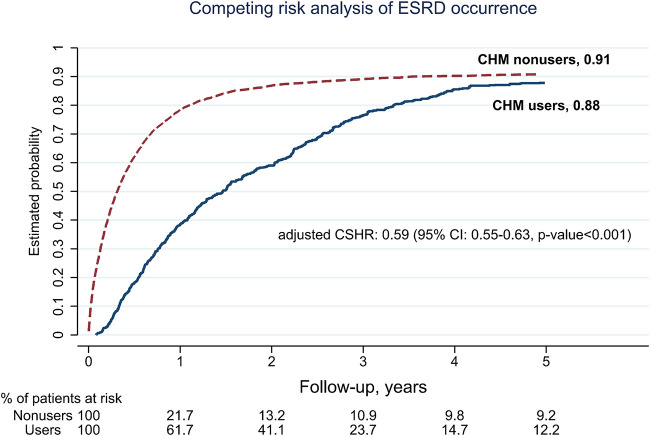
5-year follow-up for the occurrence of end-stage of renal disease (ESRD) among pre-dialysis diabetic nephropathy (DN) patients by using competing risk analysis after balancing covariates with overlap weighting (Abbreviations: CSHR, cause-specific hazard ratio; CI, confidence interval).

**FIGURE 4:**
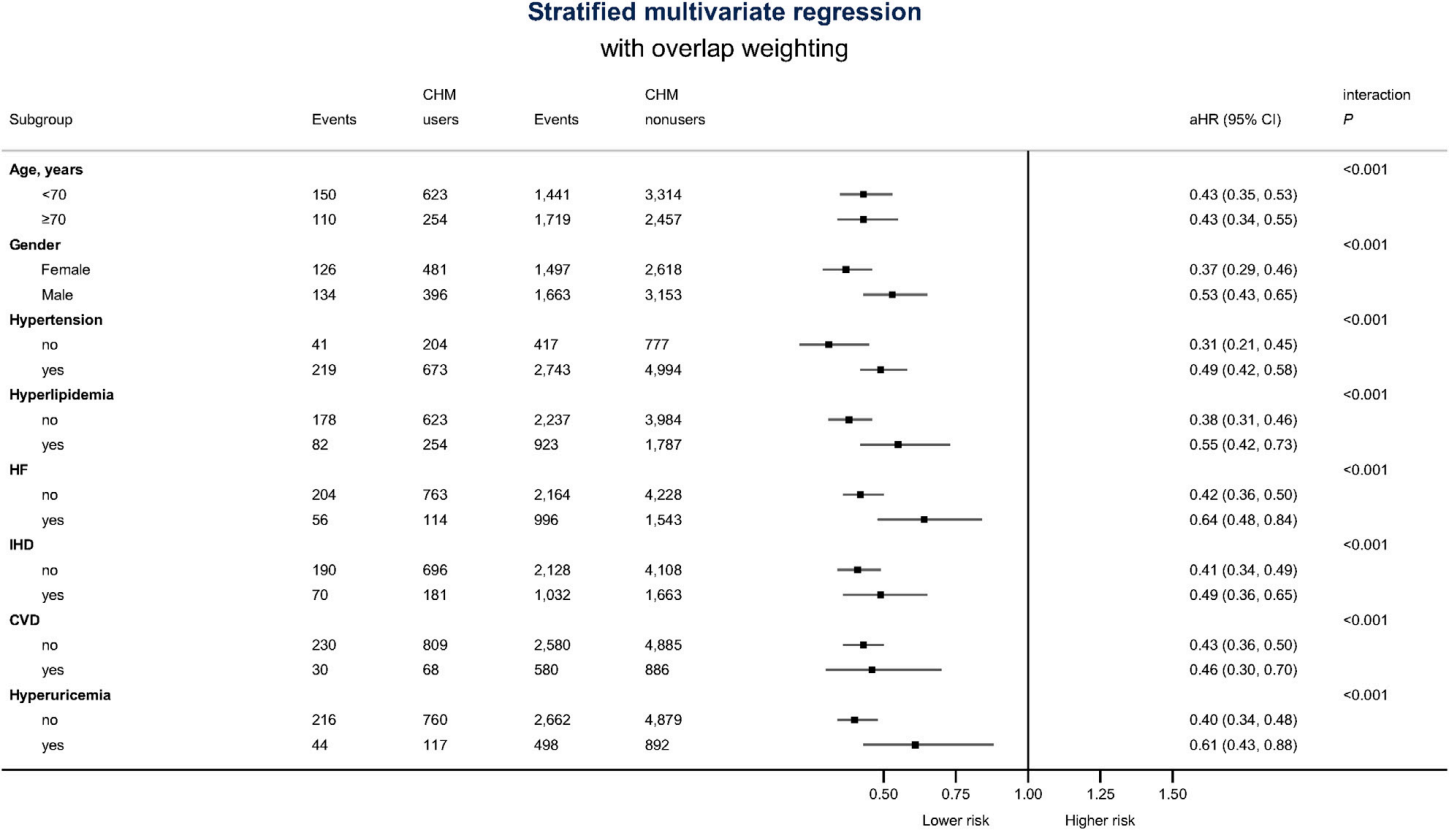
Forest plot of stratified multivariate analysis.

Besides, CHM users were associated with a lower probability for ESRD during the 5-year follow-up period. There were 775 CHM users, and 5,238 CHM nonusers finally entered the ESRD (Figure 1). The median time to ESRD occurrence was 1.14 for CHM users and 0.32 for CHM nonusers (*p*-value < 0.001). Using competing risk analysis with covariate balancing, the estimated probability of the ESRD occurrence was 0.88 for CHM users and 0.91 for CHM nonusers (Figure 3). The cause-specific hazard ratio (CSHR) for CHM users was 0.59, with adjusting covariates in the competing risk regression model (95% CI: 0.55–0.63, *p*-value <0.001). The association between CHM use and lower mortality seemed consistent in the sensitivity tests. We examined the results using a 1:1 propensity score matching and IPTW method with the same covariates used in the overlap weighting, 180-day landmark analysis, and analysis on different populations (Table 3). Although the subjects were not the same in the different models, the risks of mortality reduction seemed more prominent among CHM users than patients with all kinds of TCM modalities (aHR for CHM users: 0.42, 95%CI: 0.36–0.49 versus 0.71 for subjects with all kinds of TCM modalities, 95%CI: 0.67–0.74; Tables 2,3, respectively). Besides, the same associations could be found in the stratified multivariate regression across the subjects separate by age, gender, hypertension, hyperlipidemia, heart failure, CAD, CVD, and hyperuricemia (Figure 4).

**TABLE 3:** Sensitivity tests.

	HR (95% CI)	*p*-value	aHR (95% CI)	*p*-value
Different covariates matching/weighting methods				
1:1 PSM (*n* = 1,744)	0.59 (0.50–0.69)	<0.001	0.55 (0.47–0.65)	<0.001
Inverse probability of treatment weights	0.69 (0.59–0.80)	<0.001	0.63 (0.54–0.73)	<0.001
Models with different populations with overlap weighting				
Model with 180-days landmark analysis (*n* = 6,392)	0.32 (0.28–0.37)	<0.001	0.46 (0.40–0.54)	<0.001
Model with finally analyzed subjects (*n* = 6,648), combined with patients died in 90 days after recognition of pre-dialysis status and patients used CHM less than 30 days (*n* = 7,101)	0.29 (0.25–0.33)	<0.001	0.44 (0.38–0.51)	<0.001
Model with finally analyzed subjects, combined with users with other TCM modalities (*n* = 16,625)	0.57 (0.54–0.59)	<0.001	0.71 (0.67–0.74)	<0.001

*Abbreviations: aHR, adjusted hazard ratio; CHM, Chinese herbal medicine; CI, confidence interval; HR, hazard ratio; PSM, propensity score matching.*

### Chinese Herbal Medicine Network (CMN) for Pre-dialysis DN Patients

**FIGURE 5:**
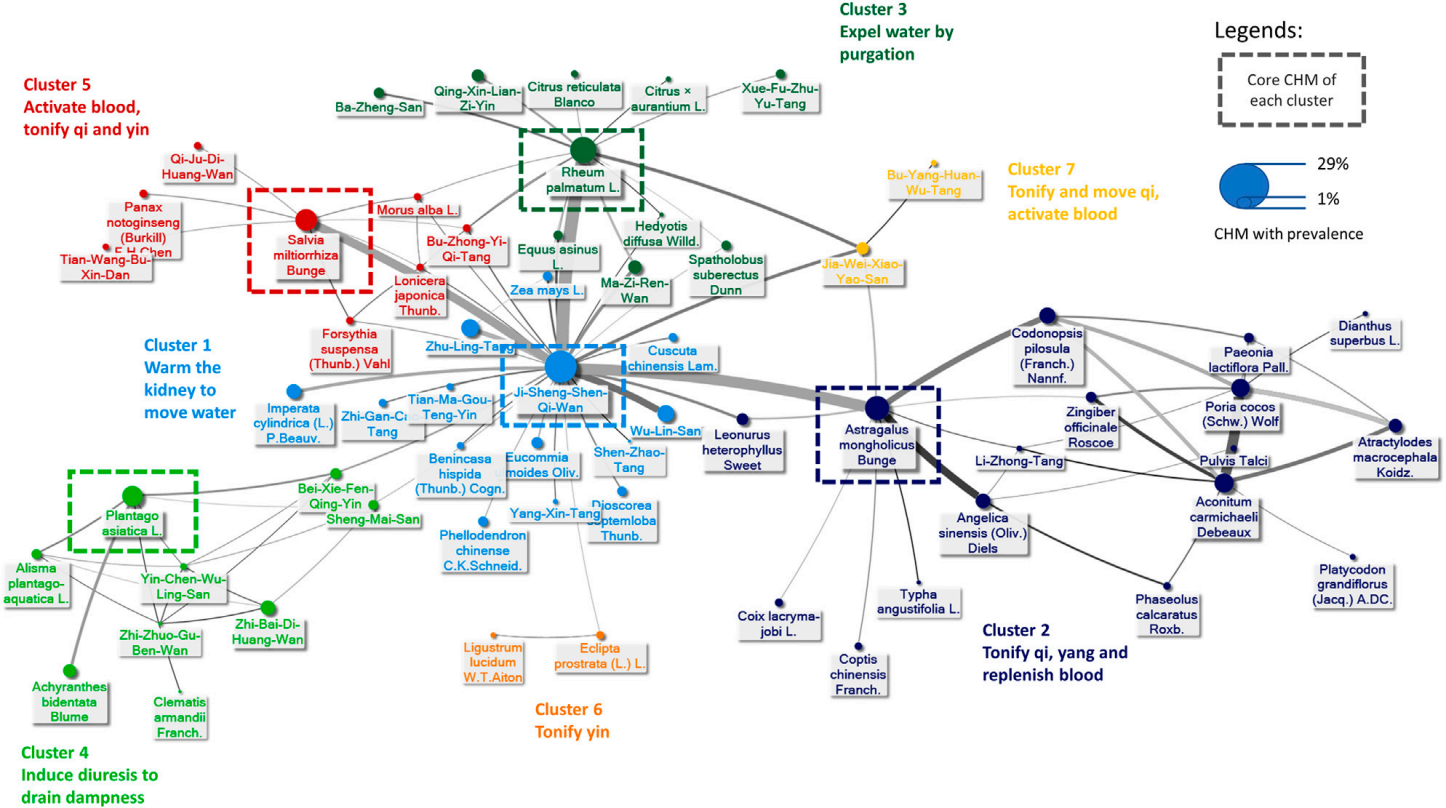
Graphic demonstration of the Chinese herbal medicine (CHM) for pre-dialysis DN patients. The connections between every CHM formed the Chinese herbal medicine network (CMN), in which thicker and darker lines means higher prevalence and stronger connections. CHMs with closer relationships were gathered into different clusters, presented by using different colors. The full name of CHMs is listed in Supplementary File S2, and the composition of herbal formula (HF) is listed in Supplementary File S3.

**FIGURE 6:**
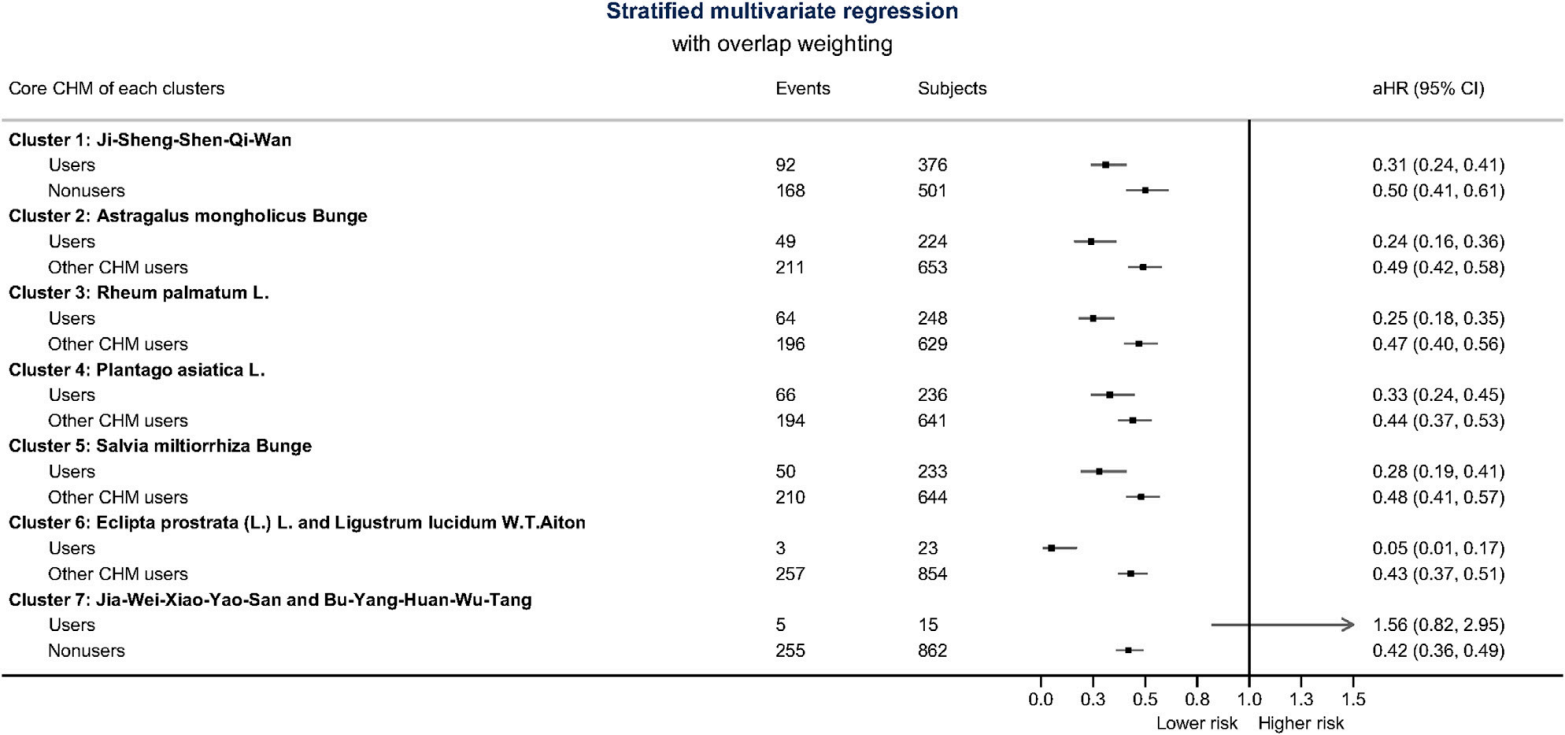
The associations of using core Chinese herbal medicine (CHM) or other CHMs with all-cause mortality reference to CHM nonusers. Generally, subjects using core CHM with every cluster had lower risks. Cluster 6 and 7 are presented as two CHMs in combination rather than core CHM since there were only two CHMs in clusters 6 and 7. The composition of herbal formula (HF) is listed in Supplementary File S3.

A total of 5,901 prescriptions were prescribed with 531 kinds of CHMs, and 5.9 CHMs were used in each prescription on average (standard deviation: 2.8). The top 100 prevalent CHM combinations were used to construct CMN, and SNA was done to reveal the core CHMs (Figure 5). CHM combinations used for CMN are listed in the Supplementary File S1, and the CHMs of each cluster are listed in Supplementary File S2. The composition of HF in the network is listed in the Supplementary File S3. CHM with larger circles meant higher prevalence in the CMN, thicker connecting lines represented higher prescription frequency, and darker connection lines indicated closer relations between connected CHMs. By incorporating CHM indications with CMN, seven CHM clusters could be found as warming kidney to remove water, tonifying qi and yang, purgating to expel water, draining dampness by diuresis, facilitating blood circulation, toxifying yin, and last but not least, toxifying and activating qi with blood (Figure 5). Ji-Sheng-Shen-Qi-Wan, *Astragalus mongholicus* Bunge (or *Astragalus membranaceus* (Fisch.) Bge)*, Plantago asiatica* L. (or *Plantago depressa* Wild.), *Salvia miltiorrhiza* Bunge, and *Rheum palmatum* L. (or *Rheum tanguticum* (Maxim. ex Regel) Balf., *Rheum officinale* Baill.) were the core CHMs in different clusters due to their high prevalence and degree centrality within clusters (Table 4). The possible pharmacologic mechanisms were summarized in Table 4 (last assessed date: 2020/8/1). Moreover, subjects using core CHMs seemed to have associations to lower mortality compared to subjects without using core CHMs (Figure 6).

**TABLE 4:** The core Chinese herbal medicine (CHM) and the potential pharmacologic mechanisms for pre-dialysis diabetic nephropathy (DN) patients.

Cluster	Name	Prevalence (%)	Degree centrality	Possible mechanisms
1	Ji-sheng-shen-qi-wan	29.2	29	Preventing or delaying the onset of diabetes mellitus (Hirotani et al., 2013), and ameliorate insulin resistance via nitric oxide pathway (Hu et al., 2010)
2	*Astragalus mongholicus* bunge (or *Astragalus membranaceus* (fisch.) bge.)	17.2	10	Ameliorates renal interstitial fibrosis (Wang et al., 2015b; Shan et al., 2016; Zhou et al., 2017)
				Autophagy, including anti-oxidation, anti-inflammation and anticancer (Shan et al., 2019)
				Induce cell apoptosis and cell cycle arrest (Tay et al., 2019)
				Repair function by activating the Nrf2-Keap1 signaling pathway and inhibiting inflammation (Han et al., 2019)
3	*Rheum palmatum* L. (or *Rheum tanguticum* (maxim. Ex regel) balf. Or *Rheum officinale* baill.)	19.7	15	Attenuate autophagy and renal fibrosis (Tu et al., 2017)
				Anti-inflammatory, antitumor, antioxidant, antifibrosis and nephroprotective activities via MAPK and P13 K-AKT pathway (Sun et al., 2016)
				Controlling blood glucose and renal protective effects (Hamzeh et al., 2014)
4	*Plantago asiatica* L. (or *Plantago depressa* willd.)	12.3	6	Not known
5	*Salvia miltiorrhiza* bunge	13.9	8	Inhibit high glucose induced renal tubular epithelial cell fibrosis (Wang et al., 2015a; Jiang et al., 2016a; Jiang et al., 2016b; Xu et al., 2016; Cao et al., 2017; Nie and Li, 2018)
				Induce cell cycle arrest in renal cell carcinoma (Wei et al., 2012; Chen et al., 2017)

## Discussion

This is the first study to report the feasibility of using CHM among incident pre-dialysis DN patients to the best of our knowledge. Previous studies showed that ACEi, ARB (Wu et al., 2013), and ketoanalogue supplementation are beneficial to pre-dialysis patients (Wu et al., 2017). For example, the PREPARE-2 study showing a reduced lower mortality risk of both dual RAS blockade and single ACEi usage (Voskamp et al., 2017). Nevertheless, no study in Chinese herbal medicine treating pre-dialysis DN patients were explored. Our study result indicated that even in the pre-dialysis population, the use of CHM during the pre-dialysis stage is positively associated with lower all-cause mortality rate during a 5-year follow-up period, which was consistent with previous studies about the general CKD or DM population (Hsu et al., 2014a; Chen et al., 2019). Our target population is similar to the previous research, with 85.2% of all patients having hypertension and 62.9% taking ACEi/ARB, suggesting that CHM might be feasible for pre-dialysis DN patients. The strict prohibition on CHM with possible renal toxicity, such as Aristolochia acid, from 20 years ago in Taiwan may also improve the safety of using CHM (Jhuang et al., 2019). However, clinical trials were expected to further solid proof for CHM protecting renal function and reduce the mortality rate.

Trying to lower the mortality rate of DN patients, especially in pre-dialysis patients, is of great importance (Hsu et al., 2016). The prevalence and incidence of dialysis are high in Taiwan, while DM accounted for about 40% of ESRD. In 2014, the DM population in Taiwan had reached 2.2 million, and the all-cause mortality rate of DM patients was 2.2 and 3.28% in female and male, respectively, making it 2.6 and 3.2 years shorter in life expectancy compared to the entire population if DM was diagnosed at the age of 40-year-old (Li et al., 2019b). That would be a significant loss given that the DM population base is enormous. Combining the positive findings for incident pre-dialysis DN patients in this study and our previous work about positive associations between TCM users among all incident DN patients, the considerable potential of using CHM among DN patients could be exploited.

The delayed course to ESRD among CHM users may be one of the causes of lower all-cause mortality. First of all, among the DM patients, the mortality rate of those under renal replacement therapy is not only higher than the average population (Saran et al., 2020) but even higher during the first few months initiating renal replacement therapy, especially in the elderly patients, no matter peritoneal dialysis or hemodialysis (Khan et al., 1995; Rosansky et al., 2011; Robinson et al., 2014). Moreover, the famous IDEAL study, a sizable randomized-controlled trial (RCT), recruiting patients from 32 centers throughout Australia and New Zealand from 2000 to 2008, shows that early initiation of dialysis treatment would not improve survival (Cooper et al., 2010). A study from Park et al. showed a consistent result that starting dialysis earlier does not improve clinical outcomes in pre-dialysis patients aging over 65-year-old (Park et al., 2017). Many researchers reviewed this issue during the past two decades, even suggested that early initiation, while the GFR is not low enough, would increase the mortality rate (Liberek et al., 2011; Rosansky et al., 2011; Peng et al., 2019). A study that reviewed over 300,000 patients starting dialysis from 1996 to 1999 suggested an increased risk of death if starting the dialysis therapy at a higher eGFR level, despite the high or low risk of the populations (Kazmi et al., 2005). Though there is no definite GFR cut point of when should dialysis initiated, the 2014 Canadian Society of Nephrology advocates to treat symptoms and signs before eGFR declines below 15 ml/min/1.73 m^2^ and commence dialysis therapy when eGFR below 6 ml/min/1.73 m^2^ or when symptoms occur (Nesrallah et al., 2014). The 2015 KDOQI guidelines did not emphasize starting dialysis according to the eGFR; instead, it stresses the observation of uremic signs and symptoms and evidence of protein-energy wasting, metabolic abnormalities, or volume overload (National Kidney, 2015). Therefore, when CHM may potentially postpone the starting time of dialysis, it is perhaps not only beneficial to maintaining GFR but also relieving the symptoms.

From the analysis on the CMN from prescriptions made for pre-dialysis DN patients, we can find the core CHM for these patients, in which the importance could be proven by subgroup analysis based on CHM users with or without using core CHMs and the potential pharmacology mechanisms. Seven clusters were identified in our analysis. The first cluster with core CHM of Ji-Sheng-Shen-Qi-Wan made a difference in reducing all-cause mortality risk of 69% compared to the other CHM, which reduced 50% risk. Ji-Sheng-Shen-Qi-Wan was proved to have the effect of delaying the onset of diabetes mellitus (Hirotani et al., 2013), and ameliorate insulin resistance via the nitric oxide pathway (Hu et al., 2010). In this cluster, the other two significantly related CHM were Zhu-Ling-Tang and Wu-Lin-San, which served to remove dampness and promote urination while correcting Ying and Yang in the body. *In vivo* study also showed that Wu-Lin-San reduced nuclear factor-kB, transforming growth factor-beta, and fibronectin accumulation in the kidney of diabetic rats induced by streptozotocin (Liu et al., 2009).

The second cluster with core CHM of *Astragalus mongholicus* Bunge (or *Astragalus membranaceus* (Fisch.) Bge.) reduced 76% of risk compared to 51% for the other CHM. Studies reported that *Astragalus mongholicus* Bunge reduced inflammation and prevented renal interstitial fibrosis (Wang et al., 2015b; Shan et al., 2016; Zhou et al., 2017), regulated cell apoptosis and cell cycle arrest (Tay et al., 2019) and last but not least, it promoted repair function by activating the Nrf2-Keap1 signaling pathway and inhibiting inflammation (Han et al., 2019). The third cluster with core CHM of *Rheum palmatum* L (or *Rheum tanguticum* (Maxim. ex Regel) Balf. or *Rheum officinale* Baill.)*,* had aHR of 0.25 while the other CHM was 0.47. *Rheum palmatum* L. was reported to modulate MAPK and P13K-AKT pathway (Hamzeh et al., 2014; Sun et al., 2016) that reduced inflammation and renal fibrosis (Tu et al., 2017). The study also suggested that *Rheum officinale* Baill. reduced expression of transforming growth factor beta 1 (TGF-β1), connective tissue growth factor (CTGF), and α-SMA in rats, possibly by preventing the declination of L-carnitine and L-acetylcarnitine in plasma of chronic renal failure rats (Zhang et al., 2016). Moreover, the fourth cluster with core CHM of *Plantago asiatica* L*.* or *Plantago depressa* Wild. lessen 67% risk while the other CHM showed 56%. Though no direct study of *Plantago asiatica* L. related to DN was done, other studies showed that it exerted an anti-hyperuricemic effect (Xia et al., 2017) and helped regulate cholesterol, triglyceride, low-density lipoprotein cholesterol, and free fatty acid in obese and metabolic abnormal mice induced by high-fat diet (Yang et al., 2017). The fifth cluster with core CHM of *Salvia miltiorrhiza* Bunge had aHR of 0.28, and the other CHM was 0.48. Studies suggested that *Salvia miltiorrhiza* Bunge inhibits high glucose-induced renal tubular epithelial cell fibrosis by regulating TGF-β/Smad expression, NF-κB signaling pathway, and glycogen synthase kinase (GSK)3β overactivity (Wang et al., 2015a; Jiang et al., 2016a; Jiang et al., 2016b; Xu et al., 2016; Cao et al., 2017; Nie and Li, 2018). Since cardiovascular events are closely related to mortality and morbidity in diabetes patients, CHM beneficial to cardiovascular system may be helpful, such as *Salvia miltiorrhiza* Bunge exerting cardiovascular protective activity (Wang et al., 2017) on blood pressure and atherosclerosis.

Our study's advantages are the authentic nature and relatively large sample size, which is essential for pre-dialysis patients since randomized-controlled trials may not be feasible and efficiently designed for pre-dialysis patients. Nonetheless, the use of CHMs for pre-dialysis patients should still be cautioned and prescribed by certificated TCM doctors or practitioners even though we reported the CMN for pre-dialysis DN. Herbal toxicity, not only renal toxicity, should still be noticed (Hudson et al., 2018; Yang et al., 2018; Enioutina et al., 2020). An example is aconitine, a suspicious compound related to kidney toxicity (Yang et al., 2018). Aconitine could be found in Ji-Sheng-Shen-Qi-Wan, the core CHM and commonest CHM used in the pre-dialysis stage, and *Aconitum carmichaeli* Debeaux, one of the SH commonly used with other CHM in CMN cluster 2. But our results showed use of Ji-Sheng-Shen-Qi-Wan-based prescription may be more beneficial than prescriptions without Ji-Sheng-Shen-Qi-Wan, and this fact reflected the importance of studying CHM combinations. Additionally, there were reports about hypokalemic nephropathy induced by glycyrrhetinic acid (a compound contained in *Glycyrrhiza uralensis* Fisch. ex DC.), and renal interstitial fibrosis caused by anthraquinone compounds contained in *Rheum officinale* Baill. Both these two SHs were commonly used to deal with diseases. It seems that not all CHMs in CMN were undoubtedly beneficial to patients, and proper combinations of CHMs seemed crucial to achieving effectiveness. Besides, some reports showed the suspicious liver injury caused by possible drug-drug interactions between extracts of *Panax ginseng* C.A.Mey. and atorvastatin or valsartan (Laube and Liu, 2019; Jeon et al., 2020). Use of *Panax ginseng* C.A.Mey. was not uncommon for pre-dialysis DN patients, and hypertension and hyperlipidemia were both commonly seen among these patients; the side effects may also be kept in mind when using these CHMs.

There are still several limitations. First, we used the starting use of EPO as the surrogate for the pre-dialysis stage among our patients. Although the number of eligible subjects may be underestimated since not all pre-dialysis patients would receive EPO, we can select the subjects with the most precise diagnosis of the pre-dialysis stage to obtain the most approximate estimations. Secondly, though self-paid CHM is not included in the national health data, the bias should be insignificant due to the large price gap between self-paid and insurance copayment, at least more than five times the price on average (Chen et al., 2019). Third, the exact blood pressure level, glycohemoglobin level, body mass index, creatinine level, and proteinuria were not available from the NHIRD since this database has been built-up for insurance purposes. Therefore, it was impossible to make sure every subjects' initial status was similar at the laboratory examination level or validated the laboratory data results. For this issue, we used overlap weighting, IPTW, and 1:1 PSM methods to overcome the confounding bias; however, there would still be unmeasurable covariates since these propensity score models were based on demographic features, such as gender, age, socio-economic status, co-morbidities, and medications. The effects of CHM for pre-dialysis patients might still be exaggerated since there were prominent differences in baseline features of CHM users and nonusers. For example, the CHM users were younger and with fewer co-morbidities; this may be related to the inconvenience of obtaining TCM care among elderly patients since they may need other people for hospital delivery (Chen et al., 2007). This may imply poor physical or nutrition conditions among these populations and may also cause worse outcomes than younger patients; however, these factors are not available in this database. Therefore, it is essential to validate the causal relationships between CHM use and reduced mortality/ESRD risks for pre-dialysis DN patients.

## Conclusion

Our study demonstrates that compared to conventional western medicine therapy, add-on CHM management may be of considerable potential. However, more clinical evidence is still needed, especially from well-designed clinical studies. Based on the core CHM explored in this study, further clinical studies could be designed more efficiently.

## Data Availability

The original contributions presented in the study are included in the article/Supplementary Material, further inquiries can be directed to the corresponding author.

## Supplementary Material

Supplementary Table S1 | CHM combinations for CMN

Supplementary Table S2 | CHMs in each cluster

Supplementary Table S3 | composition of CHM herbal formula

## References

[B1] Bikbov B. Purcell C. A. Levey A. S. Smith M. Abdoli A. Abebe M. (2020). Global, regional, and national burden of chronic kidney disease, 1990–2017: a systematic analysis for the Global Burden of Disease Study 2017. Lancet 395, 709–733. 10.1016/S0140-6736(20)30045-3 32061315 PMC7049905

[B2] Cao L. Huang B. Fu X. Yang J. Lin Y. Lin F. (2017). Effects of tanshinone IIA on the regulation of renal proximal tubular fibrosis. Mol. Med. Rep. 15, 4247–4252. 10.3892/mmr.2017.6498 28440499

[B3] Chang S. Huang Y. Lee M. Hu S. Hsiao Y. C. Chang S. W. (2018). Association of varicose veins with incident venous thromboembolism and peripheral artery disease. J. Am. Med. Assoc. 319, 807–817. 10.1001/jama.2018.0246 PMC5838574 29486040

[B4] Chen F. P. Chen T. J. Kung Y. Y. Chen Y. C. Chou L. F. Chen F. J. (2007). Use frequency of traditional Chinese medicine in Taiwan. BMC Health Serv. Res. 7, 26. 10.1186/1472-6963-7-26 17319950 PMC1810531

[B5] Chen H. Y. Lin Y. H. Huang J. W. Chen Y. C. (2015). Chinese herbal medicine network and core treatments for allergic skin diseases: implications from a nationwide database. J. Ethnopharmacol. 168, 260–267. 10.1016/j.jep.2015.04.002 25865681

[B6] Chen H. Y. Lin Y. H. Wu J. C. Chen Y. C. Thien P. F. Chen T. J. (2012). Characteristics of pediatric traditional Chinese medicine users in Taiwan: a nationwide cohort study. Pediatrics 129, e1485-92. 10.1542/peds.2011-3008 22585761

[B7] Chen H. Y. Pan H. C. Chen Y. C. Chen Y. C. Lin Y. H. Yang S. H. (2019). Traditional Chinese medicine use is associated with lower end-stage renal disease and mortality rates among patients with diabetic nephropathy: a population-based cohort study. BMC Compl. Alternative Med. 19, 81. 10.1186/s12906-019-2491-y PMC6448220 30943956

[B8] Chen W. Chen H. Y. Yang Y. H. Yang S. H. Yang C. W. Wu Y. H. (2018). An investigation of the prescription patterns of Chinese herbal products for chronic glomerulonephritis patients: a hospital-based cross-sectional study. Evid Based Complement Alternat Med 2018, 5080764. 10.1155/2018/5080764 30581484 PMC6276402

[B9] Chen Z. Zhu R. Zheng J. Chen C. Huang C. Ma J. (2017). Cryptotanshinone inhibits proliferation yet induces apoptosis by suppressing STAT3 signals in renal cell carcinoma. Oncotarget 8, 50023–50033. 10.18632/oncotarget.18483 28654902 PMC5564825

[B10] Cooper B. A. Branley P. Bulfone L. Collins J. F. Craig J. C. Fraenkel M. B. (2010). A randomized, controlled trial of early versus late initiation of dialysis. N. Engl. J. Med. 363, 609–619. 10.1056/NEJMoa1000552 20581422

[B11] Enioutina E. Y. Job K. M. Sherwin C. M. T. (2020). Why we need to pay attention to toxicity associated with herbal medicines. Br. J. Clin. Pharmacol. 86, 1793–1794. 10.1111/bcp.14340 32406066 PMC7444788

[B12] Gregg E. W. Li Y. Wang J. Burrows N. R. Ali M. K. Rolka D. (2014). Changes in diabetes-related complications in the United States, 1990-2010. N. Engl. J. Med. 370, 1514–1523. 10.1056/NEJMoa1310799 24738668

[B13] Hamzeh S. Farokhi F. Heydari R. Manaffar R. (2014). Renoprotective effect of hydroalcoholic extract of Rheum ribes root in diabetic female rats. Avicenna J Phytomed 4, 392–401. 25386403 PMC4224953

[B14] Han J. Guo D. Sun X. Y. Wang J. M. Ouyang J. M. Gui B. S. (2019). Repair effects of *Astragalus* polysaccharides with different molecular weights on oxidatively damaged HK-2 cells. Sci. Rep. 9, 9871. 10.1038/s41598-019-46264-y 31285477 PMC6614371

[B15] Hirotani Y. Okumura K. Yoko U. Myotoku M. (2013). Effects of Gosha-jinks-gan (Chinese herbal medicine: niu-Che-Sen-Qi-Wan) on hyperinsulinemia and hypertriglyceridemia in prediabetic Zucker fatty rats. Drug Discov. Ther. 7, 105–108. 23917858

[B16] Hsieh C. Y. Su C. C. Shao S. C. Sung S. F. Lin S. J. Kao Yang Y. H. (2019). Taiwan's national health insurance research database: past and future. Clin. Epidemiol. 11, 349–358. 10.2147/CLEP.S196293 31118821 PMC6509937

[B17] Hsu P. C. Tsai Y. T. Lai J. N. Wu C. T. Lin S. K. Huang C. Y. (2014a). Integrating traditional Chinese medicine healthcare into diabetes care by reducing the risk of developing kidney failure among type 2 diabetic patients: a population-based case control study. J. Ethnopharmacol. 156, 358–364. 10.1016/j.jep.2014.08.029 25178949

[B18] Hsu R. K. Chai B. Roy J. A. Anderson A. H. Bansal N. Feldman H. I. (2016). Abrupt decline in kidney function before initiating hemodialysis and all-cause mortality: the chronic renal insufficiency cohort (CRIC) study. Am. J. Kidney Dis. 68, 193–202. 10.1053/j.ajkd.2015.12.025 26830447 PMC4967032

[B19] Hsu T. W. Liu J. S. Hung S. C. Kuo K. L. Chang Y. K. Chen Y. C. (2014b). Renoprotective effect of renin-angiotensin-aldosterone system blockade in patients with predialysis advanced chronic kidney disease, hypertension, and anemia. JAMA Intern. Med. 174, 347–354. 10.1001/jamainternmed.2013.12700 24343093

[B20] Hu X. Sato J. Bajotto G. Khookhor O. Ohsawa I. Oshida Y. (2010). Goshajinkigan (Chinese herbal medicine niu-che-sen-qi-wan) improves insulin resistance in diabetic rats via the nitric oxide pathway. Nagoya J. Med. Sci. 72, 35–42. 20229701 PMC11254373

[B21] Hudson A. Lopez E. Almalki A. J. Roe A. L. Calderón A. I. (2018). A review of the toxicity of compounds found in herbal dietary supplements. Planta Med. 84, 613–626. 10.1055/a-0605-3786 29672820

[B22] Jeon J. H. Lee S. Lee W. Jin S. Kwon M. Shin C. H. (2020). Herb-drug interaction of red ginseng extract and ginsenoside rc with valsartan in rats. Molecules 25, 622. 10.3390/molecules25030622 PMC7037682 32023909

[B23] Jhuang J. R. Chiang C. J. Su S. Y. Yang Y. W. Lee W. C. (2019). Reduction in the incidence of urological cancers after the ban on Chinese herbal products containing aristolochic acid: an interrupted time-series analysis. Sci. Rep. 9, 19860. 10.1038/s41598-019-56394-y 31882686 PMC6934535

[B24] Jiang C. Zhu W. Shao Q. Yan X. Jin B. Zhang M. (2016a). Tanshinone IIA protects against folic acid-induced acute kidney injury. Am. J. Chin. Med. 44, 737–753. 10.1142/S0192415X16500403 27222061

[B25] Jiang C. Zhu W. Yan X. Shao Q. Xu B. Zhang M. (2016b). Rescue therapy with Tanshinone IIA hinders transition of acute kidney injury to chronic kidney disease via targeting GSK3β. Sci. Rep. 6, 36698. 10.1038/srep36698 27857162 PMC5114614

[B26] Kazmi W. H. Gilbertson D. T. Obrador G. T. Guo H. Pereira B. J. Collins A. J. (2005). Effect of comorbidity on the increased mortality associated with early initiation of dialysis. Am. J. Kidney Dis. 46, 887–896. 10.1053/j.ajkd.2005.08.005 16253729

[B27] Khan I. H. Catto G. R. Edward N. Macleod A. M. (1995). Death during the first 90 days of dialysis: a case control study. Am. J. Kidney Dis. 25, 276–280. 10.1016/0272-6386(95)90009-8 7847355

[B28] Laube R. Liu K. (2019). An unwanted complement: rare case of potential liver injury induced by an interaction between ginseng and atorvastatin. Br. J. Clin. Pharmacol. 85, 1612–1613. 10.1111/bcp.13927 30980549 PMC6595312

[B29] Li A. Lee H. Y. Lin Y. C. (2019a). The effect of ketoanalogues on chronic kidney disease deterioration: a meta-analysis. Nutrients 11, 957. 10.3390/nu11050957 PMC6566830 31035482

[B30] Li F. Morgan K. L. Zaslavsky A. M. (2017). Balancing covariates via propensity score weighting. J. Am. Stat. Assoc. 113, 390–400. 10.1080/01621459.2016.1260466

[B31] Li H. Y. Wu Y. L. Tu S. T. Hwu C. M. Liu J. S. Chuang L. M. (2019b). Trends of mortality in diabetic patients in Taiwan: a nationwide survey in 2005-2014. J. Formos. Med. Assoc. 118 (Suppl. 2), S83–S89. 10.1016/j.jfma.2019.07.008 31351690

[B32] Liberek T. Warzocha A. Galgowska J. Taszner K. Clark W. F. Rutkowski B. (2011). When to initiate dialysis--is early start always better? Nephrol. Dial. Transplant. 26, 2087–2091. 10.1093/ndt/gfr181 21543652

[B33] Lin C. C. Wu Y. T. Yang W. C. Tsai M. J. Liu J. S. Yang C. Y. (2017). Angiotensin receptor blockers are associated with lower mortality than ACE inhibitors in predialytic stage 5 chronic kidney disease: a nationwide study of therapy with renin-angiotensin system blockade. PloS One 12, e0189126. 10.1371/journal.pone.0189126 29216260 PMC5720519

[B34] Lin M. Y. Chiu Y. W. Chang J. S. Lin H. L. Lee C. T. Chiu G. F. (2015). Association of prescribed Chinese herbal medicine use with risk of end-stage renal disease in patients with chronic kidney disease. Kidney Int. 88, 1365–1373. 10.1038/ki.2015.226 26244923

[B35] Liu I. M. Tzeng T. F. Liou S. S. Chang C. J. (2009). The amelioration of streptozotocin diabetes-induced renal damage by Wu-Ling-San (Helen Five Herb Formula), a traditional Chinese prescription. J. Ethnopharmacol. 124, 211–218. 10.1016/j.jep.2009.04.021 19397971

[B36] National Kidney F. (2015). KDOQI clinical practice guideline for hemodialysis adequacy: 2015 update. Am. J. Kidney Dis. 66, 884–930. 10.1053/j.ajkd.2015.07.015 26498416

[B37] Nesrallah G. E. Mustafa R. A. Clark W. F. Bass A. Barnieh L. Hemmelgarn B. R. (2014). Canadian Society of Nephrology 2014 clinical practice guideline for timing the initiation of chronic dialysis. CMAJ (Can. Med. Assoc. J.) 186, 112–117. 10.1503/cmaj.130363 24492525 PMC3903737

[B38] Nie J. M. Li H. F. (2018). Therapeutic effects of Salvia miltiorrhiza injection combined with telmisartan in patients with diabetic nephropathy by influencing collagen IV and fibronectin: a case-control study. Exp. Ther. Med. 16, 3405–3412. 10.3892/etm.2018.6654 30233688 PMC6143830

[B39] Park J. Y. Yoo K. D. Kim Y. C. Kim D. K. Joo K. W. Kang S. W. (2017). Early dialysis initiation does not improve clinical outcomes in elderly end-stage renal disease patients: a multicenter prospective cohort study. PloS One 12, e0175830. 10.1371/journal.pone.0175830 28414758 PMC5393880

[B40] Peng Y. Yang X. Chen W. Yu X. Q. (2019). Association between timing of peritoneal dialysis initiation and mortality in end-stage renal disease. Chronic Dis Transl Med 5, 37–43. 10.1016/j.cdtm.2018.10.001 30993262 PMC6449773

[B41] Robinson B. M. Zhang J. Morgenstern H. Bradbury B. D. Ng L. J. Mccullough K. P. (2014). Worldwide, mortality risk is high soon after initiation of hemodialysis. Kidney Int. 85, 158–165. 10.1038/ki.2013.252 23802192 PMC3877739

[B42] Rosansky S. J. Eggers P. Jackson K. Glassock R. Clark W. F. (2011). Early start of hemodialysis may be harmful. Arch. Intern. Med. 171, 396–403. 10.1001/archinternmed.2010.415 21059968

[B43] Saran R. Robinson B. Abbott K. C. Bragg-Gresham J. Chen X. Gipson D. (2020). US renal data system 2019 annual data report: epidemiology of kidney disease in the United States. Am. J. Kidney Dis. 75, A6–A7. 10.1053/j.ajkd.2019.09.003 31704083

[B44] Sedgwick P. (2012). What is recall bias? BMJ 344, e3519.

[B45] Shan G. Zhou X. J. Xia Y. Qian H. J. (2016). *Astragalus* membranaceus ameliorates renal interstitial fibrosis by inhibiting tubular epithelial-mesenchymal transition *in vivo* and *in vitro* . Exp Ther Med 11, 1611–1616. 10.3892/etm.2016.3152 27168780 PMC4840494

[B46] Shan H. Zheng X. Li M. (2019). The effects of *Astragalus* membranaceus active extracts on autophagy-related diseases. Int. J. Mol. Sci. 20, 1904. 10.3390/ijms20081904 PMC6514605 30999666

[B47] Singhal R. Hux J. E. Alibhai S. M. Oliver M. J. (2014). Inadequate predialysis care and mortality after initiation of renal replacement therapy. Kidney Int. 86, 399–406. 10.1038/ki.2014.16 24552848

[B48] Soldatos G. Cooper M. E. (2008). Diabetic nephropathy: important pathophysiologic mechanisms. Diabetes Res. Clin. Pract. 82 (Suppl. 1), S75–S79. 10.1016/j.diabres.2008.09.042 18994672

[B49] Suissa S. (2007). Immortal time bias in observational studies of drug effects. Pharmacoepidemiol. Drug Saf. 16, 241–249. 10.1002/pds.1357 17252614

[B50] Sun H. Luo G. Chen D. Xiang Z. (2016). A comprehensive and system review for the pharmacological mechanism of action of rhein, an active anthraquinone ingredient. Front. Pharmacol. 7, 247. 10.3389/fphar.2016.00247 27582705 PMC4987408

[B51] Tay K. C. Tan L. T. Chan C. K. Hong S. L. Chan K. G. Yap W. H. (2019). Formononetin: a review of its anticancer potentials and mechanisms. Front. Pharmacol. 10, 820. 10.3389/fphar.2019.00820 31402861 PMC6676344

[B52] Tu Y. Gu L. Chen D. Wu W. Liu H. Hu H. (2017). Rhein inhibits autophagy in rat renal tubular cells by regulation of AMPK/mTOR signaling. Sci. Rep. 7, 43790. 10.1038/srep43790 28252052 PMC5333140

[B53] Vallon V. Komers R. (2011). Pathophysiology of the diabetic kidney. Comp. Physiol. 1, 1175–1232. 10.1002/cphy.c100049 PMC6029262 23733640

[B54] Voskamp P. W. M. Dekker F. W. Van Diepen M. Hoogeveen E. K. Group P.-S. (2017). Effect of dual compared to no or single renin-angiotensin system blockade on risk of renal replacement therapy or death in predialysis patients: PREPARE-2 study. J. Am. Soc. Hypertens. 11, 635–643. 10.1016/j.jash.2017.07.006 28802945

[B55] Wang D. T. Huang R. H. Cheng X. Zhang Z. H. Yang Y. J. Lin X. (2015a). Tanshinone IIA attenuates renal fibrosis and inflammation via altering expression of TGF-β/Smad and NF-κB signaling pathway in 5/6 nephrectomized rats. Int. Immunopharm. 26, 4–12. 10.1016/j.intimp.2015.02.027 25744602

[B71] Wang L. Ma R. Liu C. Liu H. Zhu R. Guo S. (2017). ASalvia miltiorrhiza: a potential red light to the development of cardiovascular diseases. Curr. Pharm. Des. 23 (7), 1077–1097. 10.2174/1381612822666161010105242 27748194 PMC5421141

[B56] Wang Y. Lin C. Ren Q. Liu Y. Yang X. (2015b). Astragaloside effect on TGF-β1, SMAD2/3, and α-SMA expression in the kidney tissues of diabetic KKAy mice. Int. J. Clin. Exp. Pathol. 8, 6828–6834. 26261569 PMC4525903

[B57] Wei X. Zhou L. Hu L. Huang Y. (2012). Tanshinone IIA arrests cell cycle and induces apoptosis in 786-O human renal cell carcinoma cells. Oncol Lett 3, 1144–1148. 10.3892/ol.2012.626 22783408 PMC3389690

[B58] Wen C. P. Cheng T. Y. Tsai M. K. Chang Y. C. Chan H. T. Tsai S. P. (2008). All-cause mortality attributable to chronic kidney disease: a prospective cohort study based on 462 293 adults in Taiwan. Lancet 371, 2173–2182. 10.1016/S0140-6736(08)60952-6 18586172

[B59] Wu C. H. Yang Y. W. Hung S. C. Kuo K. L. Wu K. D. Wu V. C. National Taiwan University Study Group on Acute Renal Failure. (2017). Ketoanalogues supplementation decreases dialysis and mortality risk in patients with anemic advanced chronic kidney disease. PloS One 12, e0176847. 10.1371/journal.pone.0176847 28475591 PMC5419544

[B60] Wu C. Y. Chen Y. J. Ho H. J. Hsu Y. C. Kuo K. N. Wu M. S. (2012). Association between nucleoside analogues and risk of hepatitis B virus–related hepatocellular carcinoma recurrence following liver resection. J. Am. Med. Assoc. 308, 1906–1914. 10.1001/2012.jama.11975 23162861

[B61] Wu H. Y. Huang J. W. Lin H. J. Liao W. C. Peng Y. S. Hung K. Y. (2013). Comparative effectiveness of renin-angiotensin system blockers and other antihypertensive drugs in patients with diabetes: systematic review and bayesian network meta-analysis. BMJ 347, f6008. 10.1136/bmj.f6008 24157497 PMC3807847

[B62] Xia N. Li B.-A. Liu H.-J. Fan J.-B. Shen W. He X.-G. (2017). Anti-hyperuricemic effect of *Plantago depressa* Wild extract in rats. Trop. J. Pharmaceut. Res. 16, 1365–1368. 10.4314/tjpr.v16i6.21

[B63] Xu L. Shen P. Bi Y. Chen J. Xiao Z. Zhang X. (2016). Danshen injection ameliorates STZ-induced diabetic nephropathy in association with suppression of oxidative stress, pro-inflammatory factors and fibrosis. Int. Immunopharm. 38, 385–394. 10.1016/j.intimp.2016.06.024 27355131

[B64] Yang B. Xie Y. Guo M. Rosner M. H. Yang H. Ronco C. (2018). Nephrotoxicity and Chinese herbal medicine. Clin. J. Am. Soc. Nephrol. 13, 1605–1611. 10.2215/CJN.11571017 29615394 PMC6218812

[B65] Yang Q. Qi M. Tong R. Wang D. Ding L. Li Z. (2017). Plantago asiatica L. Seed extract improves lipid accumulation and hyperglycemia in high-fat diet-induced obese mice. Int. J. Mol. Sci. 18, 1393. 10.3390/ijms18071393 PMC5535886 28665305

[B66] Yang T. H. Chen H. Y. Yang S. H. Lin Y. H. Fang J. T. Hung C. C. (2014). Utilization pattern for traditional Chinese medicine among late stage chronic kidney disease patients: a hospital-based cross-sectional study. J. Chin. Med. 25 (1), 41–58. 10.3966/101764462014062501003

[B67] Zhang L. Yang L. Shergis J. Zhang L. Zhang A. L. Guo X. (2019). Chinese herbal medicine for diabetic kidney disease: a systematic review and meta-analysis of randomized placebo-controlled trials. BMJ Open 9, e025653. 10.1136/bmjopen-2018-025653 PMC6501976 31048437

[B68] Zhang Z. H. Vaziri N. D. Wei F. Cheng X. L. Bai X. Zhao Y. Y. (2016). An integrated lipidomics and metabonomics reveal nephroprotective effect and biochemical mechanism of Rheum officinale in chronic renal failure. Sci. Rep. 6, 22151. 10.1038/srep22151 26903149 PMC4763304

[B69] Zhou X. Sun X. Gong X. Yang Y. Chen C. Shan G. (2017). Astragaloside IV from *Astragalus* membranaceus ameliorates renal interstitial fibrosis by inhibiting inflammation via TLR4/NF-кB *in vivo* and *in vitro* . Int. Immunopharm. 42, 18–24. 10.1016/j.intimp.2016.11.006 27855303

